# Directed evolution and secretory expression of xylose isomerase for improved utilisation of xylose in *Saccharomyces cerevisiae*

**DOI:** 10.1186/s13068-021-02073-y

**Published:** 2021-11-25

**Authors:** Jung-Hoon Bae, Mi-Jin Kim, Bong Hyun Sung, Yong-Su Jin, Jung-Hoon Sohn

**Affiliations:** 1 Synthetic Biology and Bioengineering Research Center, Korea Research Institute of Bioscience and Biotechnology (KRIBB), 125 Gwahak-ro, Yuseong-gu, Daejeon, 34141 Republic of Korea; 2 Department of Food Science and Human Nutrition, University of Illinois at Urbana-Champaign, Urbana, IL 61801 USA; 3 Cellapy Bio Inc., Bio-Venture Center 211, 125 Gwahak-ro, Yuseong-gu, Daejeon, 34141 Republic of Korea

**Keywords:** Xylose, Xylose isomerase, Secretion, *Saccharomyces cerevisiae*, Co-fermentation

## Abstract

**Background:**

Xylose contained in lignocellulosic biomass is an attractive carbon substrate for economically viable conversion to bioethanol. Extensive research has been conducted on xylose fermentation using recombinant *Saccharomyces cerevisiae* expressing xylose isomerase (XI) and xylose reductase/xylitol dehydrogenase (XR/XDH) pathways along with the introduction of a xylose transporter and amplification of the downstream pathway. However, the low utilization of xylose in the presence of glucose, due to the varying preference for cellular uptake, is a lingering challenge. Studies so far have mainly focused on xylose utilization inside the cells, but there have been little trials on the conversion of xylose to xylulose by cell before uptake. We hypothesized that the extracellular conversion of xylose to xylulose before uptake would facilitate better utilization of xylose even in the presence of glucose. To verify this, XI from *Piromyces* sp. was engineered and hyper-secreted in *S. cerevisiae* for the extracellular conversion of xylose to xylulose.

**Results:**

The optimal pH of XI was lowered from 7.0 to 5.0 by directed evolution to ensure its high activity under the acidic conditions used for yeast fermentation, and hyper-secretion of an engineered XI-76 mutant (E56A and I252M) was accomplished by employing target protein-specific translational fusion partners. The purified XI-76 showed twofold higher activity than that of the wild type at pH 5. The secretory expression of XI-76 in the previously developed xylose utilizing yeast strain, SR8 increased xylose consumption and ethanol production by approximately 7–20% and 15–20% in xylose fermentation and glucose and xylose co-fermentation, respectively.

**Conclusions:**

Isomerisation of xylose to xylulose before uptake using extracellular XI was found to be effective in xylose fermentation or glucose/xylose co-fermentation. This suggested that glucose competed less with xylulose than with xylose for uptake by the cell. Consequently, the engineered XI secretion system constructed in this study can pave the way for simultaneous utilization of C5/C6 sugars from the sustainable lignocellulosic biomass.

**Supplementary Information:**

The online version contains supplementary material available at 10.1186/s13068-021-02073-y.

## Background

Utilisation of lignocellulosic biomass for the production of bio-based energy is an urgent issue to identify sustainable alternatives to conventional fossil fuels. Lignocellulosic biomass has been considered the most abundant carbohydrate source for bioethanol production, and it does not compete with food resources [1]. Most early studies on the utilisation of lignocellulosic biomass have focused on the fermentation of glucose derived from cellulose, which can be easily converted to ethanol. As lignocellulosic biomass contains a significant amount of hemicellulose (up to 25–50% of the total dry weight), there is a need to develop a cost-effective bioethanol production process that efficiently utilises xylose, which is the most abundant pentose sugar in hemicellulose [2, 3].

*Saccharomyces cerevisiae* has been an attractive industrial workhorse for bioethanol production owing to high ethanol yields and its general robustness to environmental stresses. However, xylose is not an easy substrate for *S. cerevisiae* due to the low uptake rate and low catabolic activity for xylose. Although some yeasts, such as *Scheffersomyces stipitis*, can utilise xylose, their ethanol productivity and stress tolerance were considerably lower than those of *S. cerevisiae* [4]. Therefore, numerous studies have focused on metabolic engineering for establishment and improvement of xylose fermentation in *S. cerevisiae* (reviewed in [5]).

Xylose assimilation is generally achieved by two different pathways. In most xylose-utilizing eukaryotes, xylose is converted into xylulose via xylitol by NADPH-dependent xylose reductase (XR) followed by an NAD^+^-dependent xylitol dehydrogenase (XDH) [6–8]. In contrast, bacteria directly convert xylose to xylulose by xylose isomerase (XI) [9–12]. The product of both pathways, xylulose is phosphorylated by xylulose kinase (XK) and then enters into the pentose phosphate pathway for conversion to ethanol. Comparison of both pathways in different *S. cerevisiae* strains using various substrates [13–16] revealed that the XI pathway always gave a higher ethanol yield than the XR/XDH pathway. When both pathways were introduced into a single *S. cerevisiae* strain, a synergistic effect on xylose fermentation was observed [17, 18]. In addition, introduction of a xylose transporter or amplification of the downstream pathway has also proven to be beneficial [19, 20].

Therefore, for the development of xylose-fermenting *S. cerevisiae*, the inclusion of both XI and XR/XDH pathways and various other modifications into a single strain is favourable.

XR/XDH should be expressed in the cytosol due to the requirement for nicotinamide cofactors. Though XI is also a cytosolic enzyme, its activity is independent of cofactors. XI is inhibited by xylitol, a by-product of the XR/XDH pathway. Therefore, XI can be expressed extracellularly for xylose-fermenting yeast to escape from xylitol inhibition and to convert xylose to xylulose before uptake. Because *S. cerevisiae* takes up xylulose more rapidly than xylose [21], XI was used in immobilized form for simultaneous isomerization and fermentation (SIF) of xylose [22, 23], or expressed on the cell surface of *S. cerevisiae* for the conversion of xylose to xylulose outside the cell [24]. Nevertheless, there have been no follow-up studies, because SIF requires a large amount of purified XI for immobilization, and a surface display system is not optimal for the functional expression of XI as it requires multimerization for its activity [25]. Therefore, complete secretion of XI by the yeast itself could be more advantageous than the use of immobilised or surface-displayed XI for xylose assimilation.

In this study, we developed a recombinant *S. cerevisiae* that secretes a pH-optimized XI capable of continuously converting xylose to xylulose in the media before cell uptake during fermentation. For this purpose, *xylA* of *Piromyces* sp. E2 [26] was engineered to shift its pH optimum from 7.0 to 5.0 to fit the optimal conditions of yeast fermentation. Then, the engineered XI was hyper-secreted into the culture medium by employing target protein-specific translational fusion partners (TFP) selected from the yeast genome-wide secretion leader library [27]. By addition of the XI secretion system developed in this study to the previously developed *S. cerevisiae* SR8 strain engineered with the XR/XDH pathway [28], ethanol could be effectively produced not only in xylose single fermentation but also in xylose/glucose co-fermentation.

## Results

### Construction of recombinant *S. cerevisiae* secreting XI from *Piromyces* sp﻿. E2

**Fig. 1:**
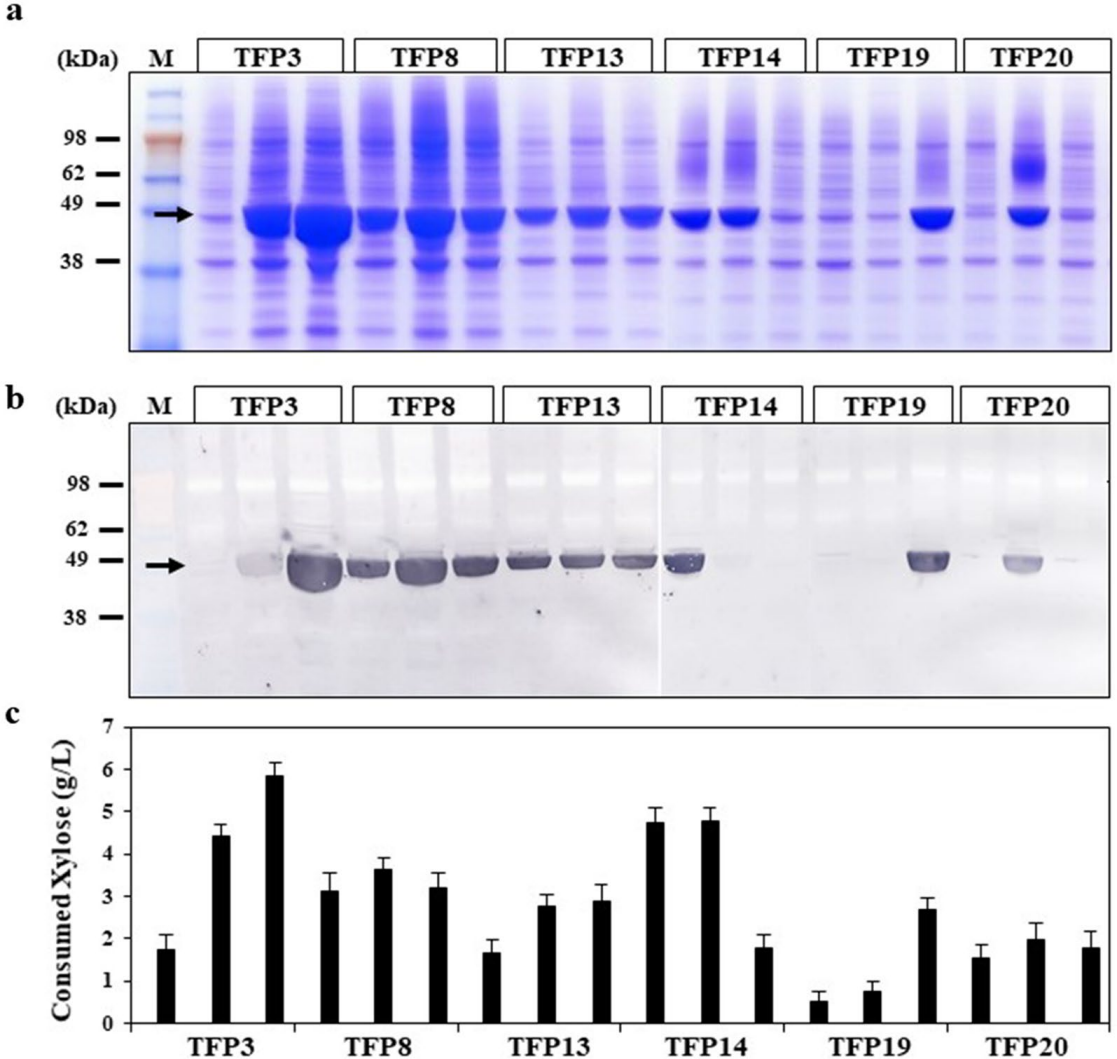
Selection of optimal translational fusion partner (TFP) for xylose isomerase (xylA) secretion in *S. cerevisiae*. **a** SDS-PAGE and **b** western blot analysis using anti-6xHIS antibody against xylA secreted by the TFP system. **c** Amount of xylose consumed after 40 h cultivation in YPDX medium. The xylA protein band is indicated by an arrow. M: protein size marker

Since the *xylA* gene from *Piromyces sp.* E2 was the first eukaryotic XI functionally expressed in *S. cerevisiae*, we selected this gene for secretory expression in *S. cerevisiae.* Given that XI is a cytosolic protein, an efficient signal peptide is required to direct this protein into the extracellular medium. The *xylA* gene was expressed under the control of *GAL10* promoter on a *URA3* selectable multi-copy vector using pre-selected 24 types of TFPs that are frequently screened for secretion of recombinant proteins [29–31]. The TFP vectors were designed to include the Kexin processing site at the junction between the TFP and *xylA* to secrete the correctly processed XI. Recombinant strains were directly constructed by co-transformation of *S. cerevisiae* 2805*Δgal80* with linearized TFP vectors and *xylA* gene flanked with linker and terminator sequences (Additional file 1: Fig. S1). After cultivation of transformants, the culture supernatants were analysed by SDS-PAGE and western blotting to compare the amount of XI secreted by the specific TFPs. Most of the TFPs secreted correctly processed XI (50 kDa) through Kexin processing but showed different secretion levels (Additional file 1: Fig. S2). Among the 24 TFPs, TFP3, 8, 13, 14, 19, and 20 showed remarkable capacity for extracellular production of recombinant XI (Additional file 1: Fig. S2). Three independent colonies were then randomly selected from each transformant and cultivated in YPDX medium containing 1% glucose and 1% xylose. The culture supernatant of each transformant was analysed by SDS-PAGE and western blotting (Fig. 1a, b). After 40 h of cultivation, the amount of xylose consumed by each transformant was compared (Fig. 1c). The amounts of secreted XI were proportional to the amounts of consumed xylose.

TFP3 containing 117 amino acids (aa) from the N-terminus of cell wall-associated mannoprotein CIS3 (alias PIR4) showed the highest capacity for secretion of XI, and a transformant expressing TFP3-XI consumed the highest amount of xylose (5.8 g/L).

### Comparison of different types of XI expression

**Fig. 2:**
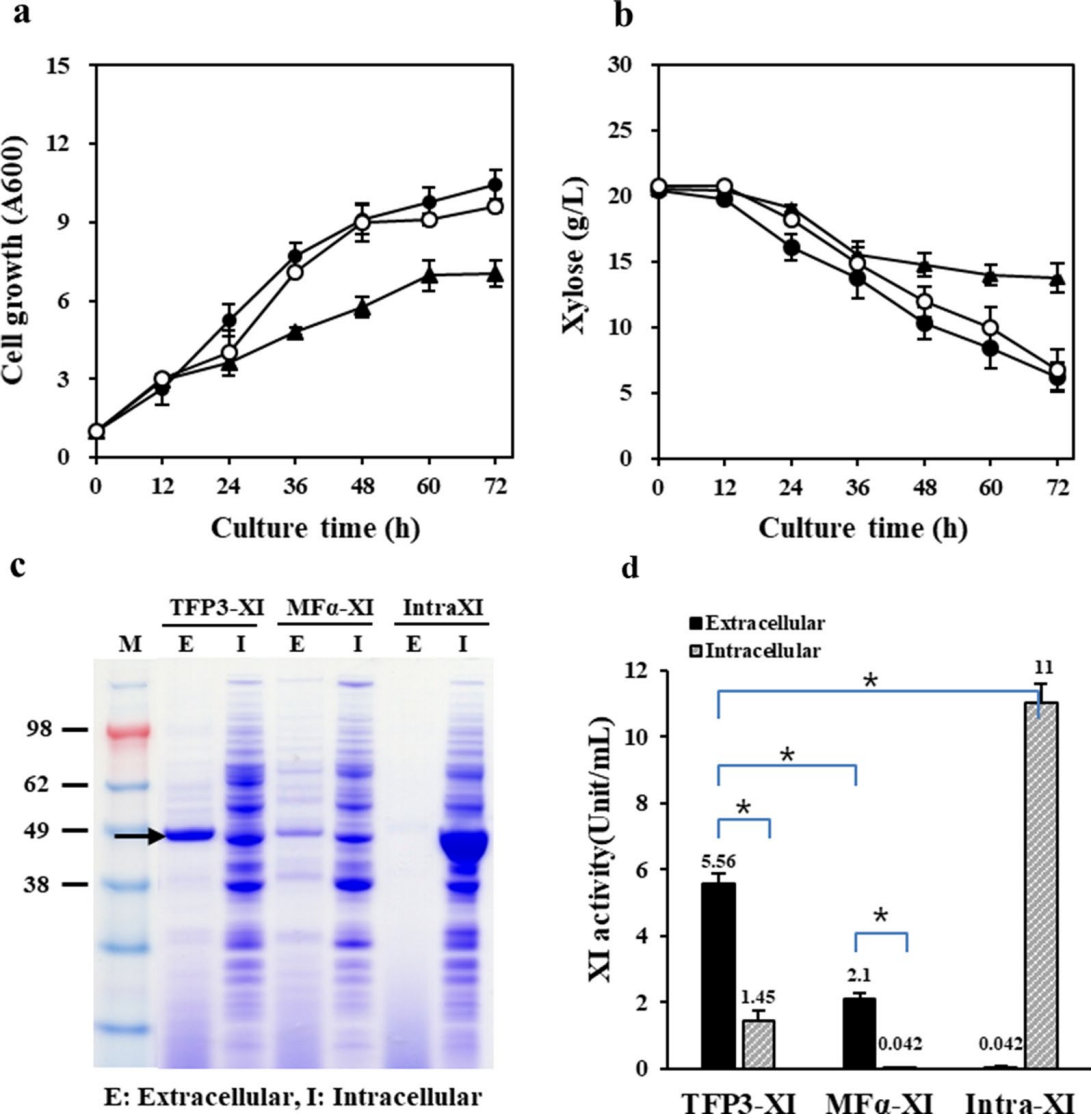
Growth and xylose consumption of three xylose-assimilating strains in YPX medium containing 2% xylose. **a** Cell growth, **b** xylose consumption, **c** SDS-PAGE and **d** XI activity of the extracellular and intracellular fractions of each strain after 72 h cultivation in YPX medium. The xylA protein band is indicated by an arrow. Mean values and standard deviations of triplicates are shown. *p < 0.03. Symbols: TFP3-XI (●), MFα-XI (▲), Intra-XI (○)

In yeast, mating factor alpha (MFα) pre–pro peptide of *S. cerevisiae* has been generally used for secretion of recombinant proteins because of its outstanding qualifications [32]. To confirm the secretion-enhancing effect of TFP3, growth and xylose consumption of transformants expressing TFP3-XI, MFα-XI, and intra-XI (intracellular XI) were compared in YPX medium containing 2% xylose as a sole carbon source, and the extracellular and intracellular fractions of each transformant were analysed after 72 h of cultivation (Fig. 2). Each transformant expressing TFP3-XI and intra-XI showed 40% and 80% of enhanced growth and xylose consumption compared to those of the transformant expressing MFα-XI at the end of fermentation (Fig. 2a, b). The amount and activity of secreted XI using TFP3 were twice those achieved using MFα signal peptide (Fig. 2c, d). Since the XI from *Piromyces* sp﻿. E2 is a cytosolic protein, no XI protein was detected in the extracellular fraction of a transformant expressing intra-XI, but the total XI activity (sum of intra and extracellular activity) was higher than that of the transformant expressing TFP3-XI (Fig. 2d). Despite showing the highest total XI activity, the growth and xylose consumption of the intra-XI expressing strain were similar to those of the strain expressing TFP3-XI. These results imply that extracellular conversion of xylose is more desirable than intracellular conversion for xylose utilization.

### Characterisation of secretory expressed XI

**Fig. 3:**
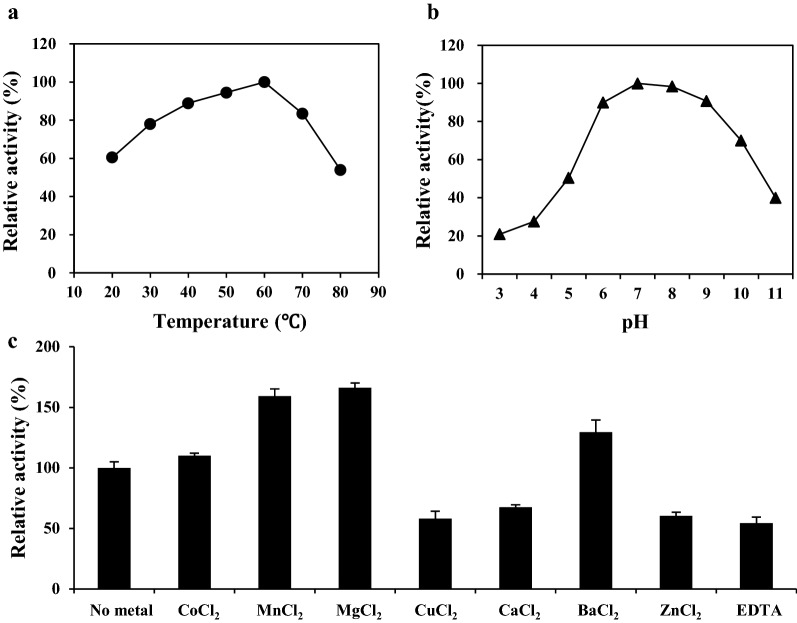
Characterization of extracellularly produced recombinant xylose isomerase. Effects of **a** temperature, **b** pH, and **c** metal ions on XI activity were determined by quantitative analysis of xylulose with HPLC

For the secretory production of XI, fed-batch fermentation of recombinant *S. cerevisiae* 2805*Δgal80* strains expressing TFP3-XI was performed, and recombinant XI was purified from the culture broth using Ni-affinity chromatography. The optimum temperature of the purified XI was analysed in 50 mM Tris–HCl buffer (pH 7.5) and the optimum pH was analysed at 60 °C (Fig. 3). The activity of purified XI increased steadily with an increase in temperature until 60 °C and then decreased rapidly. The purified XI showed narrow optimum pH at neutral condition. As XI is a multimeric enzyme requiring two bivalent metal ions, the effect of various metal ions on the XI activity was confirmed. Contrary to the XI expressed in *E. coli* [33], the XI produced in yeast showed a slightly higher preference for Mg^2+^ than Mn^2+^ (Fig. 3c). The two metal ions are involved in binding of the substrate and the isomerisation reaction [34]. Therefore, optimum concentrations of metal ions required for the efficient utilisation of xylose were determined by comparing growth of *S. cerevisiae* 2805*Δgal80* strains expressing TFP3-XI in YPX medium containing various concentrations of MgCl_2_ and MnCl_2_ (Additional file 1: Fig. S3). The *S. cerevisiae* transformant showed saturated growth at 0.5 mM MnCl_2_ and highest growth at 20 mM MgCl_2_. When these two ions were used simultaneously, growth was further increased by approximately 30% compared to the case where MnCl_2_ was used alone. Therefore, 0.5 mM MnCl_2_ and 20 mM MgCl_2_ were added to the medium hereafter.

### Insufficient conversion of xylose to xylulose by secreted XI

**Fig. 4:**
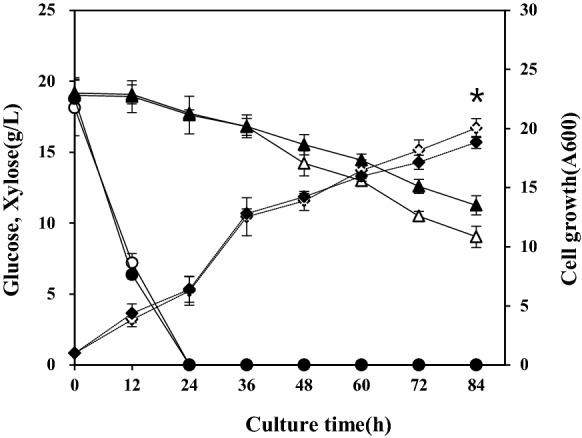
Fermentation of two strains expressing intracellular XI and extracellular XI. YPDX medium containing 2% glucose and 2% xylose was used for fermentation. Mean values and standard deviations of triplicates are shown. *p < 0.03. Symbols: TFP3-XI: Glucose (○), Intra-XI: Glucose (●), TFP3-XI: Xylose (△), Intra-XI: Xylose (▲), TFP3-XI: Growth (◊), Intra-XI: Growth (◆)

To assess the consumption of xylose in the presence of glucose, transformants expressing TFP3-XI and intra-XI were cultured in flasks containing YPDX medium (2% glucose and 2% xylose) for 84 h, and growth and the amount of xylose consumed were analysed (Fig. 4). After 36 h of fermentation, the TFP3-XI strain showed enhanced xylose consumption and growth compared to the intra-XI strain (Fig. 4). However, to our disappointment, the xylose was almost not used in the early part of fermentation when glucose was also present. After glucose was exhausted, both strains began to use xylose slowly. This result implies that extracellular conversion of xylose to xylulose was not sufficient to support growth, or the uptake of converted xylulose was still hindered by glucose. To validate the simultaneous utilisation of xylulose and glucose, *S. cerevisiae* 2805*Δgal80* and *S. cerevisiae* 2805*Δgal80, XKS1* strains were cultured in YPDXu medium (2% glucose and 2% xylulose) for 60 h under ethanol fermentation conditions. *Gal80* mutant was used for the constitutive expression of XI under the control of a strong *GAL10* promoter without the induction of galactose [35]. Glucose utilisation was similar in both strains, but xylulose utilisation was dependent on the overexpression of XK. *S. cerevisiae* 2805*Δgal80* strain utilised xylulose after exhaustion of glucose, while *S. cerevisiae* 2805*Δgal80, XKS1* strain efficiently utilised xylulose and glucose at similar rates, albeit slightly delayed (Additional file 1: Fig. S4). Consequently, the slow utilization of xylose (Fig. 4), can be mainly attributed to the inefficient extracellular conversion of xylose to xylulose and weak xylulose assimilation of the host strain. Therefore, it is necessary to increase the extracellular activity of XI and to reinforce the downstream pathway of xylulose utilization.

### Directed evolution of XI to generate low pH-optimized variant

**Fig. 5:**
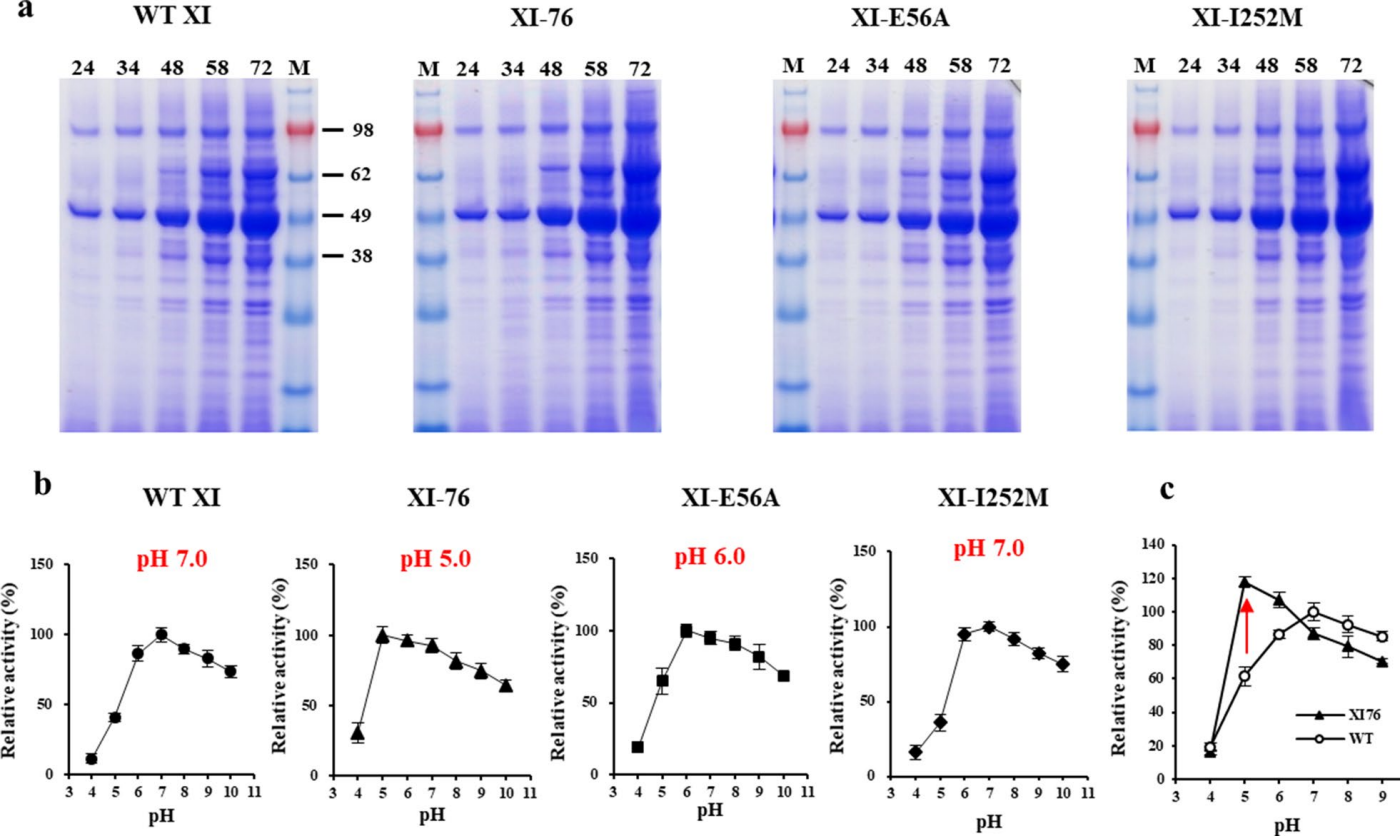
Comparison of XI variants. **a** SDS-PAGE analysis of the culture supernatant of xylose-assimilating strains expressing four kinds of XIs. Each strain was cultured in YPX medium containing 2% xylose, and 0.6 mL of culture supernatant at the indicated times was analysed after precipitation. **b** Confirmation of optimal pH of the mutant XIs. **c** Comparison of the relative activity of the purified wild type XI and XI-76. The xylA protein band is indicated by an arrow. Mean values and standard deviations of triplicates are shown

As the native XI showed only 50% activity under the culture conditions of yeast (pH 5.0) compared to that at its optimal condition (pH 7.0) (Fig. 3b), an XI mutant showing higher activity at pH around 5 is required to enhance extracellular conversion of xylose to xylulose during yeast fermentation. For this purpose, we first compared the growth of transformants secreting XI at various pH conditions. To clarify the XI-dependent growth on xylose medium, *S. cerevisiae* 2805*Δgal80* strain was used for expression of TFP3-XI instead of the downstream pathway-amplified strain (*S. cerevisiae* 2805*Δgal80, XKS1*). *S. cerevisiae* 2805*Δgal80* strain was transformed with TFP3-XI expression vector, and equal number of cells were plated on the UDX solid medium (pH 3–7) containing 0.1% glucose and 2% xylose. A small amount of glucose was added to support the initial expression of recombinant XI. As shown in Additional file 1: Fig. S5, the transformant growth was seriously dependent on the media pH. The number of transformed cells at pH 5 was less than 3% of cells at pH 6, and fewer colonies were formed at pH 4. This condition (UDX, pH 4) was used to screen low-pH optimized XI. Random mutations were generated into the XI gene (*xylA*) by error-prone PCR. Recombinant *S. cerevisiae* 2805*Δgal80* strains expressing XI mutant library were directly constructed by co-transformation of the PCR-amplified XI gene and linearized YGaTFP3 vector. Approximately 4 × 10^4^ transformants (2 × 10^3^ transformants × 20 plates) were screened on UDX solid media (pH 4.0), and hundreds of early formed colonies were transferred to 96 deep-well plates containing YPX broth (pH 4.0). Seven rapidly growing cells were selected and confirmed again by cultivation in shake flask containing 50 mL YPX (pH 4.0). The directed evolution process is summarised in Additional file 1: Fig. S6. Finally, a strain showing 36% and 20% enhanced xylose consumption and growth, respectively, compared to those of wild type, was selected, and the XI nucleotide sequence was determined. This mutant was named XI-76 according to the clone number. The XI-76 mutant contains two amino acid changes (E56A, I252M). To verify the effects of these mutations on XI activity, two mutants (XI-E56A, XI-I252M) each containing a single amino acid change were constructed. Three strains harbouring these mutations, along with the wild type, were cultured in flasks containing 50 mL YPX medium for 72 h. The amounts of XI secreted during fermentation were compared by SDS-PAGE, and the optimum pH of the mutant XIs was analysed using 72 h culture supernatants (Fig. 5). While there was no significant difference of secreted XI yield between the mutants and wild type, a clear difference was observed in the optimum pH. Between the two single mutants, only XI-E56A showed a change of optimum pH to pH 6. However, the optimum pH of the double mutant, XI-76, was synergistically changed to pH 5 by incorporation of the I252M mutation. Cell growth and xylose consumption of the strains expressing the single mutant XIs were increased by approximately 10% and further increased in XI-76 by over 20% compared to those in wild type (Table 1). To compare the relative activities of wild-type XI and XI-76 in different pH conditions, the two proteins were purified and specific activities of the purified XIs were analysed (Fig. 5c). Similar to the result obtained using crude XI, the optimum pH of purified XI-76 was changed to pH 5, and the relative activity of XI-76 was twofold higher than that of the wild-type XI at pH 5. The maximum activity of XI-76 at pH 5 was also 20% higher than that of wild-type XI at pH 7. Consequently, XI-76 could be useful for extracellular conversion of xylose to xylulose under the culture conditions of yeast *S. cerevisiae*.

**Table 1:** Cell growth and xylose consumption of strains expressing different xylose isomerase (XI) variants

Strain	Mutations	Growth (OD_600_)	Consumed xylose (g)	Optimal pH
WT	–	15.3 ± 0.5	9.0 ± 0.3	7.0
XI-76	E56A, I252M	18.6 ± 0.7	13.4 ± 0.5	5.0
XI-E56A	E56A	16.5 ± 0.5	10.8 ± 0.4	6.0
XI-I252M	I252M	16.3 ± 0.4	10.6 ± 0.4	7.0

Data shown represent the mean from triplicate experiments ± SD

### Low-pH optimized XI for xylose fermentation and glucose/xylose co-fermentation

**Fig. 6:**
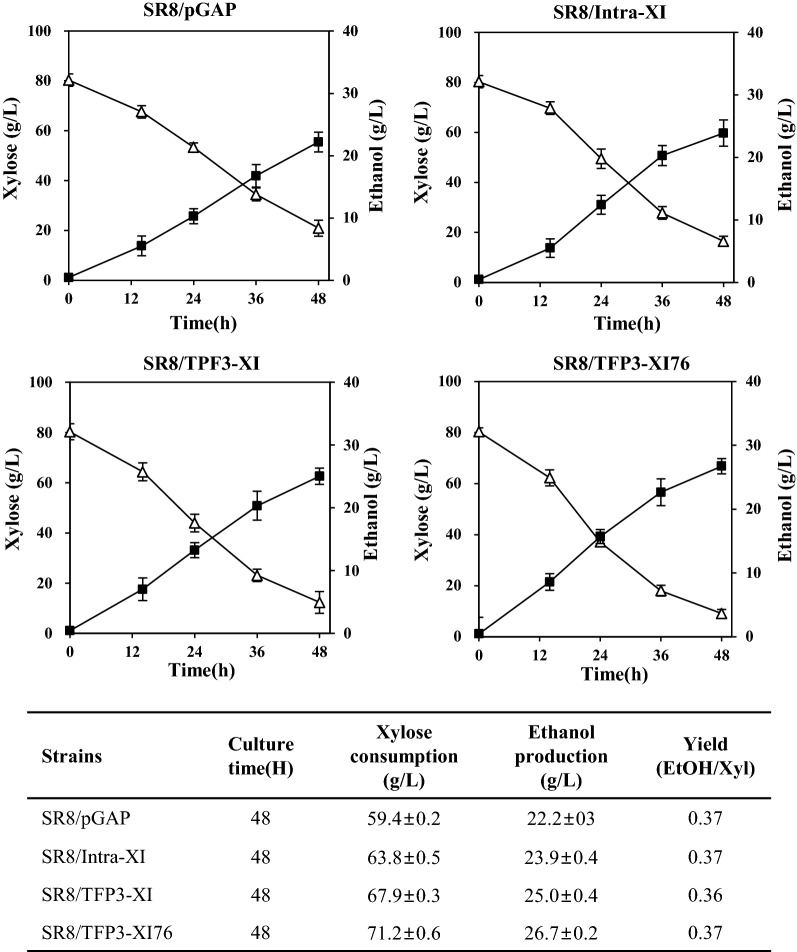
Fermentation profiles of xylose-assimilating strains expressing different XIs and an empty vector. YPX medium containing 8% xylose was used as fermentation medium. Mean values and standard deviations of triplicates are shown. Symbols: Xylose (△), Ethanol (■)

**Fig. 7:**
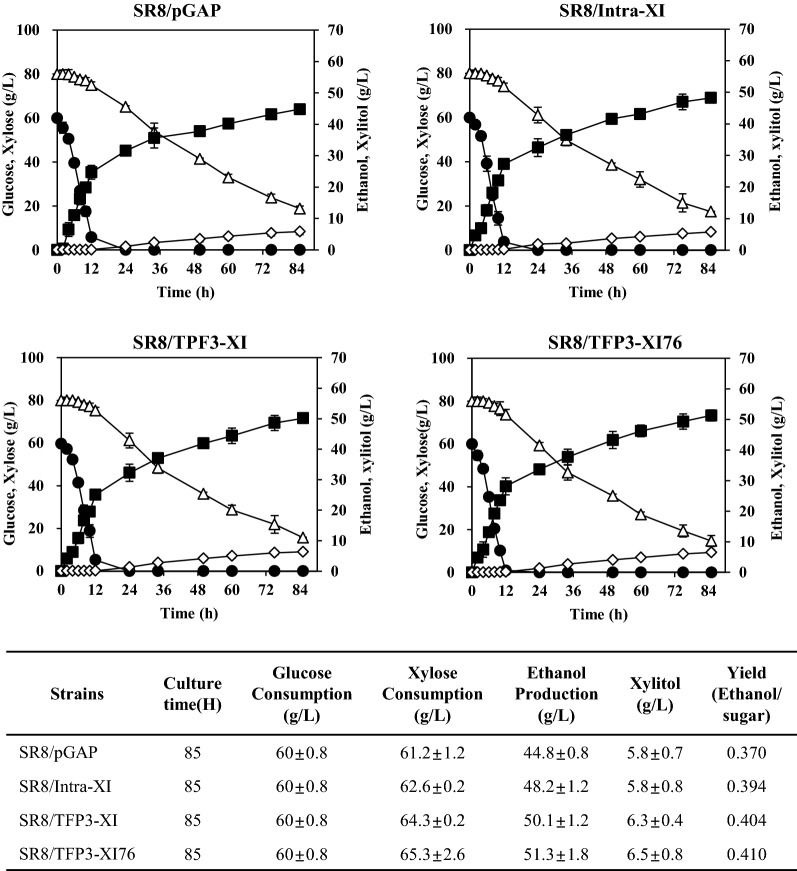
Co-fermentation profiles of xylose-assimilating strains expressing different XIs and an empty vector. YPDX medium containing 6% glucose and 8% xylose was used as fermentation medium. Mean values and standard deviations of triplicates are shown. Symbols: Glucose (●), Xylose (△), Ethanol (■), and Xylitol (◇)

To test the effect of low-pH optimized XI secretion on xylose fermentation and glucose/xylose co-fermentation, we employed a previously developed strain, *S. cerevisiae* SR8 which attained substantially improved xylose fermentation capability by optimisation of the expression levels of *XYL1*, *XYL2*, and *XYL3*, and disruption of *PHO13* and *ALD6* [28]. There was an obvious improvement of xylose consumption and ethanol yield of *S. cerevisiae* 2805*Δgal80, XKS1* strain when low-pH optimized XI was secreted, unfortunately, however, the final titer of ethanol was much lower than that of SR8 strain. Thus, we decided to introduce the low-pH optimized XI into the SR8 strain and checked the effect of XI secretion for the further improvement of xylose fermentation and glucose/xylose co-fermentation. Three strains expressing intra-XI, TFP3-XI, and TFP3-XI-76 under the control of *GAPDH* promoter and a strain containing the empty vector (pGAP) were cultured in YPX medium containing 8% xylose for 48 h. As shown in Fig. 6, the intracellular expression of XI increased xylose consumption and ethanol production by approximately 7–8% compared to the SR8 strain harbouring the empty vector. Meanwhile, a 12–13% increase in the xylose consumption and ethanol production was achieved by secretion of wild-type XI, and further increased over 20% by secretion of the XI-76 mutant. For the glucose/xylose co-fermentation, these strains were cultured in YPDX medium containing 6% glucose and 8% xylose (Fig. 7). As a result of co-fermentation for 85 h, glucose was rapidly consumed within 15 h in all strains, and the initial xylose consumption rate was insignificant between the strains, but showed a difference from 24 h when sufficient XI was secreted. The overall xylose consumption was slightly reduced compared to the case of using xylose alone, but a similar improvement was confirmed when XI-76 was secreted. Consequently, secretory expression of low-pH optimized XI-76 improved the xylose consumption and ethanol production of the strain SR8 by approximately 7% and 15% from the co-fermentation of glucose and xylose. The final ethanol yield of glucose/xylose co-fermentation was also improved from 0.37 g/g of SR8 to 0.41 g/g under the xylitol yield of 0.1 g/g xylose.

## Discussion

In this study, we developed a recombinant *S. cerevisiae* hyper-secreting XI from *Piromyces* sp﻿. E2 using the TFP screening system [36] for the extracellular conversion of xylose to xylulose. For the effective secretion of a cytosolic protein-like XI, a powerful secretion leader that drives a cargo protein into the secretion pathway is generally required. Yeast TFP library was generated from a genetic pool of yeast secretion proteins, which are composed of a signal peptide and a pro-peptide acting as secretion enhancer by assisting the folding and trafficking of cargo proteins. Among the 24 TFPs tested, TFP3 showed the highest level of XI secretion. TFP3 consists of 117 aa of the N-terminus of the CIS3 protein*.* CIS3 has a pre–pro type secretion signal processed by Kex2p. The secretion signal of CIS3 has been used as a fusion partner for the production of xylanase and lipase in *S. cerevisiae* [37, 38]. TFP3 induced the secretion of twice the amount of XI than that achieved using the MFα signal peptide, which is the most effective and popular pre–pro signal peptide in yeast.

When the cell growth and xylose consumption of the strain expressing TFP3-XI were compared with those of the strains expressing MFα-XI and intra-XI in xylose medium, the TFP3-XI strain showed 40% enhanced cell growth and xylose consumption compared to the MFα-XI strain (Fig. 2a, b), and the intra-XI strain was slightly less effective than the TFP3-XI strain. The extracellular and total XI activities of the TFP3-XI strain were 2.6- and 3.2-fold higher than that of the MFα-XI strain, respectively. This is considered to have caused the enhanced cell growth and xylose consumption of TFP3-XI. Meanwhile, the intra-XI strain showed twofold higher XI activity than TFP3-XI, but cell growth and xylose consumption were rather poor compared to TFP3-XI, demonstrating that the extracellular conversion of xylose to xylulose is more effective for xylose utilization. These results are consistent with previous reports that *S. cerevisiae* takes up xylulose better than xylose (21).

XIs have been divided into class I and class II enzymes based on their structure and metal ion preference. *Piromyces* XI expressed in *E. coli* was reported to show higher preference for Mn^2+^ compared to Mg^2+^ (29% of Mn^2+^), and consequently classified into class II [33]. However, the XI produced in this study showed a slightly higher preference for Mg^2+^ than Mn^2+^ (Fig. 3c). This difference could be due to the concentration of the metal ions used. The relative activity of XI expressed in *E. coli* was analysed at a metal ion concentration of 100 μM, while the XI expressed in yeast was analysed at 100-fold higher concentrations of the metal ions (10 mM). When these two ions were used simultaneously, a growth-improving synergy was identified at 0.5 mM MnCl_2_ and 20 mM MgCl_2_.

Optimum temperature and pH of the recombinant XI secreted from *S. cerevisiae* in this study were found to be 60 °C and pH 7–8, similar to those reported for most XI at 60 °C and pH 7–9, respectively [39]. Most studies on XI expression in *S. cerevisiae* for xylose assimilation have focused on cytosolic expression. As the cytosolic pH of *S. cerevisiae* is slightly below 7, the activity of intracellular XI is usually optimal [11]. However, as found in this study, XI activity rapidly decreased at acidic pH, with only 50% activity under the yeast culture condition (pH 5) compared to that at its optimal condition. Therefore, to enhance the extracellular conversion of xylose to xylulose under the yeast culture condition, the optimal pH of XI was shifted to near 5 by directed evolution. A variant *xylA* (XI-76) conferring enhanced xylose consumption and growth of host strain in xylose medium at pH 4 was selected. Two amino acid substitutions (E56A, I252M) identified in this mutant were verified to contribute to the shift in optimal pH of XI and work synergistically. The shift in optimal pH mainly resulted from the E56A mutation and was enhanced by the I252M mutation. The XI-76 variant exhibited a twofold increase of the specific activity at pH 5, and the maximum activity of XI-76 variant at pH 5 was over 20% higher than that of wild-type XI at pH 7. This suggested that the mutations identified in XI-76 changed the optimum pH and increased the specific activity of XI simultaneously. Directed evolution of *Piromyces* sp. XI has been previously attempted to improve xylose fermentation in *S. cerevisiae* [40, 41]. In the former study, two amino acids located near the monomer binding contacts (E15D) and the active site (T142S) were shown to be responsible for the improved XI performance, while in the latter, substitution of residues around the substrate- and metal-binding sites was explored. Although the mutations identified in the current study were not directly related to the active site, the E56A mutation clearly affected the optimal pH of XI-76 mutant. Previously, homo-tetrameric structure of *Piromyces* sp. E2 XI was determined [33] and E56 was identified on the monomer binding contact surface. When the Glu (pI 3.08) is changed to Ala (pI 6.11), the net charge of this residue is changed to positive at pH 5. Therefore, it can be inferred that positive charge at the 56th residue is more desirable for formation of multimeric XI or alterations in monomer interactions may affect substrate binding. A similar shift in the pH profile by modifying surface amino acids has been demonstrated in subtilisin [42].

To verify additional improvement of xylose fermentation, we introduced the low-pH optimized XI in a previously developed xylose-fermenting yeast *S. cerevisiae* SR8 [28, 43]. Secretory expression of XI-76 increased xylose consumption and ethanol yield by up to 20% and 12% compared to those of SR8 and SR8 strain expressing intracellular XI in YPX medium containing 8% xylose, respectively. Moreover, similar improvement was obtained with co-fermentation of glucose and xylose. Thus, xylose fermentation was definitely enhanced by the secretion of evolved XI through the extracellular conversion of xylose to xylulose. Our strain demonstrated a higher ethanol yield from mixed sugars (0.41 g/g) than the reported strains harbouring combined XR/XDH and XI pathways (0.34–0.35 g/g) [17, 18]. Nevertheless, it is still far from the theoretical maximum yield of 0.51 g/g. As previously reported, the formation of xylitol must be a reason for lowering the ethanol yield, but as confirmed in this study, the xylitol yield was not serious at around 0.1 g/g xylose. Accordingly, further optimization of other conditions will be required for the improvement of ethanol yield from xylose. Adaptive evolution of the current strain in different concentrations of glucose and xylose is also underway to enhance the ethanol yield. In addition, further improvement of ethanol yield could be expected by development of a mesophilic XI for yeast fermentation or by employing prokaryotic *xylA* gene from *Clostridium phytofermentans*, which was shown to perform better than *Piromyces* sp. XI [18].

## Conclusion

In this study, we engineered the XI from *Piromyces* sp. E2 to shift its pH optimum to favour better xylose utilization under the acidic culture conditions used for yeast fermentation. Xylose was rapidly converted into xylulose during fermentation through the low-pH optimized extracellular XI to facilitate influx into the cell. Secretory expression of the engineered XI-76 variant in *S. cerevisiae* increased xylose consumption and ethanol production by approximately 7–20% and 15–20% in xylose fermentation and glucose and xylose co-fermentation, respectively. The low-pH optimized XI secretion system developed in this study could lay a foundation for the utilization of xylose or glucose/xylose obtained from lignocellulogic biomass.

## Materials and methods

### Strains and plasmids

*Escherichia coli* DH5α [F-*lac*Z*Δ* M15 *hsd*R17(r-m-) *gyr*A36] was used for general recombinant DNA techniques. *S. cerevisiae* 2805*Δgal80* (*Mat* α *pep4::HIS3 prb1, can1, his3-200, gal80)* [35] and *S. cerevisiae* SR8 (*MATa, leu2, his3, ura3, can1, XYL1, XYL2, XKS1,* evolutionarily engineered in xylose-containing media, *ald6*) [28] were used as general hosts for expression of XI. *S. cerevisiae* 2805*Δgal80, XKS1* containing multi-copy integrated *XKS1* under the control of glyceraldehyde 3-phosphate dehydrogenase (*GAPDH*) promoter was used for the directed evolution of XI. For multi-copy integration of the *XKS1* gene into *S. cerevisiae* 2805*Δgal80,* plasmid δISXK donated by Prof. J. H. Seo was used [44]. For the construction of the pGAP-T3-*xylA* plasmid, *GAL10* promoter of YGaT3xylA was replaced with the *GAPDH* promoter amplified with GAP-F/GAP-R primers (forward 5′*-*AGAGCTCGGTACCCATCAAGCTTACCAGTTCTCACAC-3′ and reverse 5′*-*AGGATCCGTTTGTTTATGTGTGTTTATTCGA*-*3′) after digestion with *Sacl*/*Bam*HI. For construction of the pGAP-xylA﻿ plasmid, *xylA* gene amplified using intraXI-F (5′-AGGATCCATGGCAAAGGAATA-3′) and intraXI-R primers (5′-AGTCGACTTAGTGATGGTGAT-3′) was digested with *Bam*HI/*Sal*I and then cloned into the same site of pGAP-T3-xylA﻿ vector.

### Culture and analysis of secreted proteins

Yeast transformation was performed using the lithium acetate method [45]. Yeast was aerobically cultivated at 30 °C in synthetic defined medium lacking uracil (SD-ura; 0.67% yeast nitrogen base without amino acid, 2% glucose, 0.77 g/L-uracil dropout supplement, and 2% agar) for selection of transformants. YPD medium (1% Yeast Extract, 2% Peptone and 2% glucose), YPX medium (1% Yeast Extract, 2% Peptone, 2% xylose, 0.5 mM MnCl_2_ and 20 mM MgCl_2_), and YPDX medium (1% Yeast Extract, 2% Peptone, 1% glucose, 1% xylose, 0.5 mM MnCl_2_, and 20 mM MgCl_2_) were used for the expression of XI. UDX medium (0.67% yeast nitrogen base without amino acid, 0.77 g/L-uracil dropout supplement, 0.1% glucose, 2% xylose, 1 μg/mL antimycin) was used for the selection of transformants in directed evolution of XI. Transformants were cultivated in a 10-mL test tube containing 2 mL YPD broth for 40 h. Then, 0.6 mL of culture supernatant was precipitated with 0.4 mL cold acetone and analysed by electrophoresis on 12% polyacrylamide gels under denaturing conditions by staining with Coomassie blue. Western blot analysis was performed using anti-His antibody (Sigma Chemicals Co., St. Louis, MO, USA) after 1:1000 dilution. Protein samples were electrophoresed and transferred to a nitrocellulose membrane using iBot^®^ 2 Dry Blotting System (Thermo Fisher Scientific, Waltham, MA, USA) following the manufacturer’s instructions. The reacting antibodies were detected with anti-mouse immunoglobulins conjugated to alkaline phosphatase (Sigma Chemicals Co.)

### Construction of recombinant yeast secreting XI

The nucleotide sequence of *xylA* from *Piromyces* sp﻿. E2 (CAB76571.1) was codon-optimised using the codon optimisation tool from the IDT website (http://www.idtdna.com/CodonOpt), and overlapping sequences for recombinational cloning were added to the 5′ and 3′ ends of the optimised *xylA* gene. The optimised *xylA* gene was synthesized by Bioneer (Daejeon, Korea). The *xylA* gene was amplified with primers (forward LNK39 5′*-*GGCCGCCTCGGCCTCTGCTGGCCTCGCCTTAGATAAAAGA*-*3′ and reverse GT50R 5′-GTCATTATTAAATATATATATATATATATTGTCACTCCGTTCAAGTCGAC*-*3′) to expand the overlapping sequences as described previously [27]. Recombinant *S. cerevisiae* 2805*Δgal80* strains expressing *xylA* gene with TFPs were directly constructed by co-transformation of the amplified *xylA* gene and 24 linearized TFP vectors (Additional file 1: Fig. S1). Circular topology of the plasmid was restored in host cells by homologous recombination of a linearized TFP vector with *xylA* gene containing overlapping sequences at each end [46].

### Production and characterisation of XI

Fed-batch fermentation was carried out in a 5-L fermenter (KoBiotech Co., Incheon, Korea). Seed culture for fermentation was prepared in a 250-mL Erlenmeyer flask containing 50 mL of SD-ura broth at 30 °C. After 24 h growth, the seed culture was transferred into 250 mL YPD broth to prepare the pre-culture. After 24 h growth, the pre-culture was inoculated into a 5-L jar fermenter containing 1.8 L main culture medium (2% glucose, 3% yeast extract, and 1.5% peptone). The culture conditions were 30 °C and pH 5.5 maintained by NH_4_OH. During fermentation, the feeding medium (300 g/L of glucose and 50 g/L of yeast extract) was added to maintain the concentration of the glucose. The cell mass and secreted protein were analysed by optical density (OD_600_) and SDS-PAGE, respectively. The culture supernatant was filtered with 0.2-μm Sartoclear^®^ S9-P Cap (Sartorius AG, Göttingen, Germany) and concentrated tenfold by ultrafiltration with a 30 K NMWC pore size filter (GE Healthcare, Chicago, IL, USA) and 20 mM Tris–HCl (pH 7.5) buffer. The concentrated protein was applied onto an Ni–NTA affinity chromatography column (GE Healthcare) pre-equilibrated with buffer A [20 mM Tris–HCl (pH 7.5) and 0.5 M NaCl]. After washing with the washing buffer [20 mM Tris–HCl (pH 7.5), 0.5 M NaCl, and 20 mM imidazole], the bound proteins were eluted with buffer B [20 mM Tris–HCl (pH 7.5), 0.5 M NaCl, and 250 mM imidazole]. The eluted protein was concentrated using an Amicon ultra centrifugal filter and 20 mM Tris–HCl (pH 7.5) buffer. The optimum pH of the purified XI was measured at 60 °C using pH 3–11 buffers (pH 3–6: 50 mM citrate buffer, pH 6–7: 50 mM potassium phosphate buffer, pH 7–11: Tris–HCl buffer), and the optimum temperature was determined in 50 mM Tris–HCl buffer (pH 7.5) at various temperatures (20 ~ 80 °C).

To determine the effect of various metal ions on XI activity, 10 mM of various metal ion chlorides (cobalt, magnesium, manganese, cupper, calcium, barium, zinc) and ethylene diamine tetra acetic acid were pre-incubated with the enzyme solution in 50 mM Tris–HCl buffer (pH 7.5) for 15 min. After incubation, the enzyme activity was analysed at optimum condition; XI activity of the enzyme without metal ions was set to 100%. XI activity was analysed in a solution containing 50 mM Tris–HCl (pH 7.5), 10 mM MgCl_2_, and 1 mg of purified enzyme. After addition of 10% xylose, the reaction mixture was incubated at 60 °C for 3 h, and the reaction was stopped by addition of 0.1 M NaOH. The amount of xylulose produced was determined by high-performance liquid chromatography (HPLC) analysis. One unit of XI activity was defined as the amount of enzyme required to produce 1 μmol of xylulose per min under the assay conditions described above.

### Ethanol fermentation

The seed culture was prepared by incubating XI strains in test tube culture containing 10 mL YPD broth for 24 h at 30 °C with shaking at 200 rpm. Oxygen-limited fermentation was performed at 30 °C and 200 rpm in 125-mL serum bottles closed with butyl rubber stoppers and then purged with N_2_ gas using 50 mL YP medium (1% Yeast Extract, 2% Peptone, 20 mM MgCl_2_, 0.5 mM MnCl_2_) with various concentrations of glucose and xylose (2 ~ 10% glucose and 2 ~ 10% xylose). The initial cell density was adjusted to OD_600_ = 5, which was predetermined to be an optimal seed amount in a preliminary test. Glucose, xylose, xylulose, xylitol, and ethanol were analysed by HPLC and a refractive index detector (Agilent Technologies, Santa Clara, CA, USA) with an Aminex HPX-87H column (Bio-Rad, Hercules, CA, USA). The column temperature was maintained at 65 °C, and 0.005% H_2_SO_4_ was used as the mobile phase with a flow rate of 0.6 mL/min.

### Statistical analyses

All data are represented as the mean value ± standard deviation (SD) of three experiments. Statistical comparison of ethanol concentration, sugar utilized and growth were performed using the Student’s *t* test with a two-tailed distribution (Microsoft Excel) and compared to the appropriate control strain. A *p* value of < 0.05 was considered statistically significant.

## Supplementary Information

**Additional file 1: Figure S1.** Scheme for the direct construction of recombinant *xylA* expression vectors by in vivo recombination with translational fusion partners (TFPs). **Figure S2.** Optimal TFP screening for secretory expression of xylA. (a) SDS-PAGE analysis of culture broth of recombinant yeasts expressing xylA and Coomassie Blue staining. The xylA protein band was verified by western blotting analysis (b). 1–24; TFP number, M: protein size marker. **Figure S3.** Determination of optimal concentration of metal ions for growth in YPX medium containing 2% xylose as a sole carbon source. Various concentration of MnCl_2_ (a), MgCl_2_ (b) were analyzed separately and simultaneously (c). All experiment were triplicated and the results are given as mean values with error bars indicating standard deviations *p < 0.03. **Figure S4.** Co-fermentation of glucose and xylulose using wild type (a) and xylulokinase overproduced strain (b). All experiment were triplicated and the results are given as mean values with error bars indicating standard deviations Symbols: Glucose (●), Xylulose (△), Ethanol (■). **Figure S5.** Comparison of growing cells on different pH media after transformation of TFP3-XI vector. UDX solid media containing 0.1% glucose and 2% xylose with different pH from 3 to 7 were used. **Figure S6.** Summary of directed evolution of XI for the development of low-pH optimized XI.

## Data Availability

All data generated or analysed during this study are included in this published article and its Additional information files.

## Abbreviations

TFP: Translational fusion partner
SIF: Simultaneous isomerization and fermentation
XI: Xylose isomerase
XR: Xylose reductase
XDH: Xylitol dehydrogenase
XK: Xylulose kinase
