# Supplementary material for: Directed evolution and secretory expression of xylose isomerase for improved utilisation of xylose in Saccharomyces cerevisiae
Source: Biotechnol Biofuels. 2021 Nov 25;14:223. doi: 10.1186/s13068-021-02073-y (PMC8613937; doi:10.1186/s13068-021-02073-y)
Supplement: Supplementary file 1 — Additional file 1: Figure S1. Scheme for the direct construction of recombinant xylA expression vectors by in vivo recombination with translational fusion partners (TFPs). Figure S2. Optimal TFP screening for secretory expression of xylA. (a) SDS-PAGE analysis of culture broth of recombinant yeasts expressing xylA and Coomassie Blue staining. The xylA protein band was verified by western blotting analysis (b). 1–24; TFP number, M: protein size marker. Figure S3. Determination of optimal concentration of metal ions for growth in YPX medium containing 2% xylose as a sole carbon source. Various concentration of MnCl2 (a), MgCl2 (b) were analyzed separately and simultaneously (c). All experiment were triplicated and the results are given as mean values with error bars indicating standard deviations *p < 0.03. Figure S4. Co-fermentation of glucose and xylulose using wild type (a) and xylulokinase overproduced strain (b). All experiment were triplicated and the results are given as mean values with error bars indicating standard deviations Symbols: Glucose (●), Xylulose (△), Ethanol (■). Figure S5. Comparison of growing cells on different pH media after transformation of TFP3-XI vector. UDX solid media containing 0.1% glucose and 2% xylose with different pH from 3 to 7 were used. Figure S6. Summary of directed evolution of XI for the development of low-pH optimized XI. [file 13068_2021_2073_MOESM1_ESM.pptx]

## Slide 1
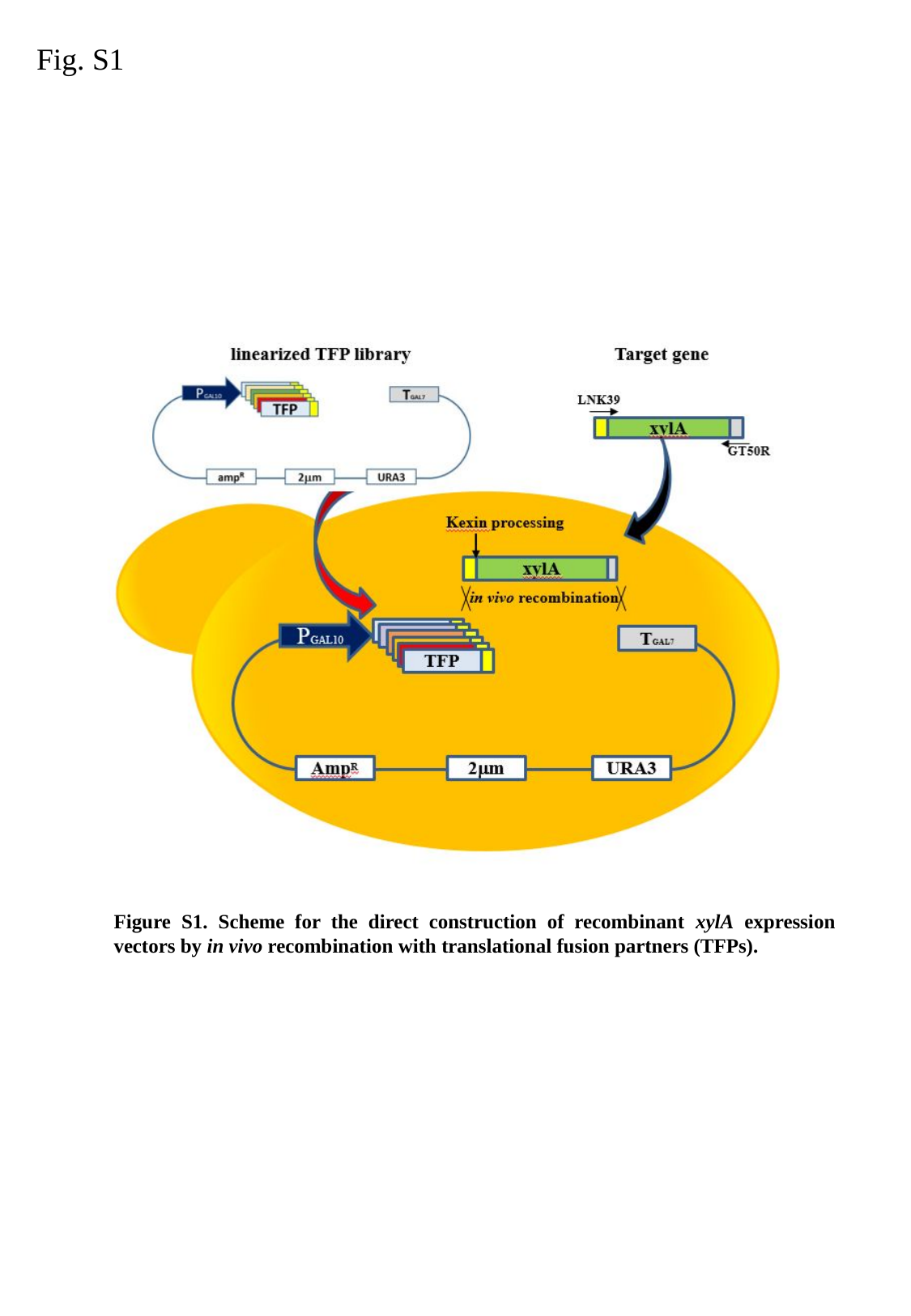

Fig. S1
Figure S1. Scheme for the direct construction of recombinant xylA expression vectors by in vivo recombination with translational fusion partners (TFPs).

## Slide 2
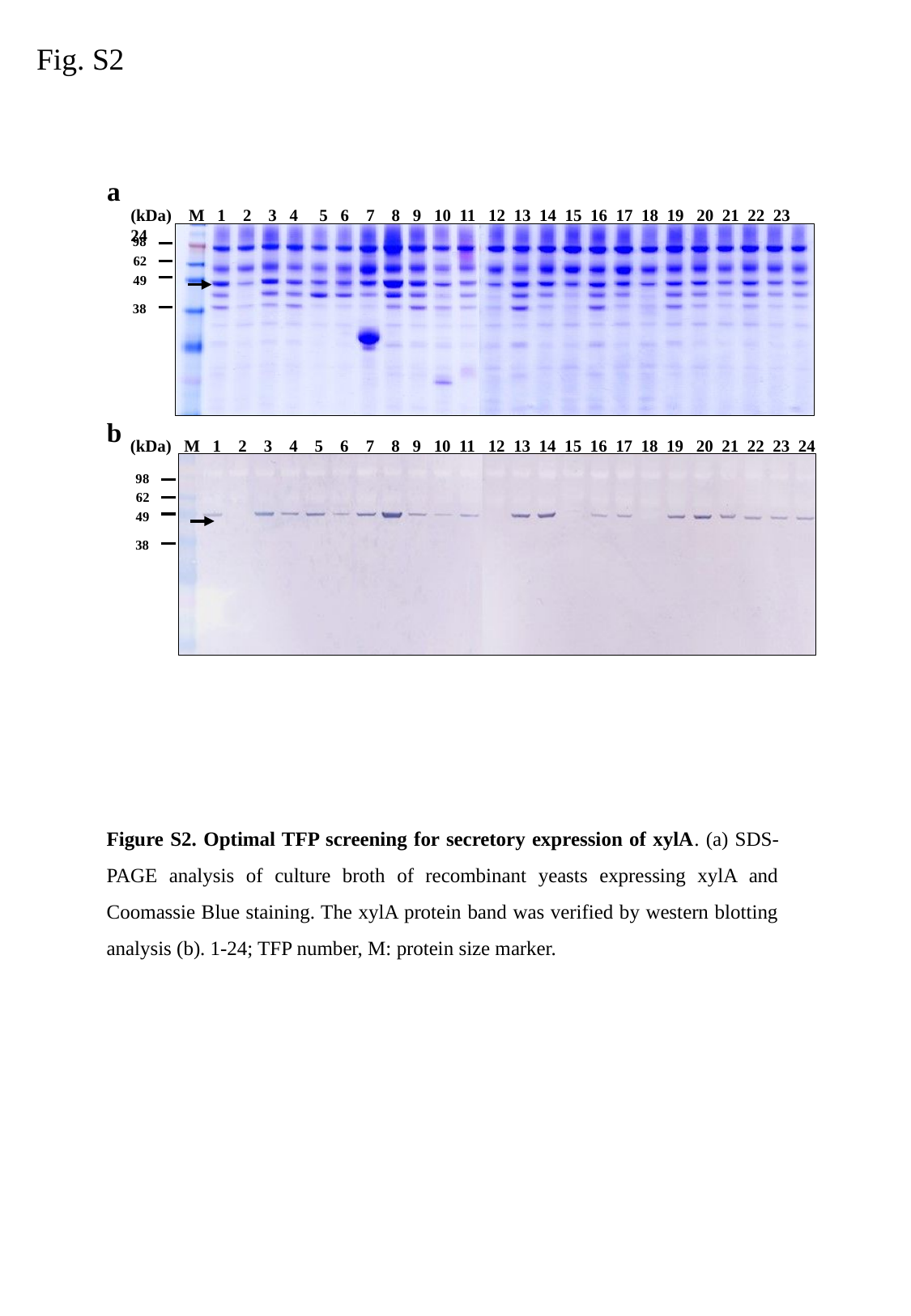

Fig. S2
a
(kDa) M 1 2 3 4 5 6 7 8 9 10 11 12 13 14 15 16 17 18 19 20 21 22 23 24
98
62
49
38
b
(kDa) M 1 2 3 4 5 6 7 8 9 10 11 12 13 14 15 16 17 18 19 20 21 22 23 24
98
62
49
38
Figure S2. Optimal TFP screening for secretory expression of xylA. (a) SDS-PAGE analysis of culture broth of recombinant yeasts expressing xylA and Coomassie Blue staining. The xylA protein band was verified by western blotting analysis (b). 1-24; TFP number, M: protein size marker.

## Slide 3
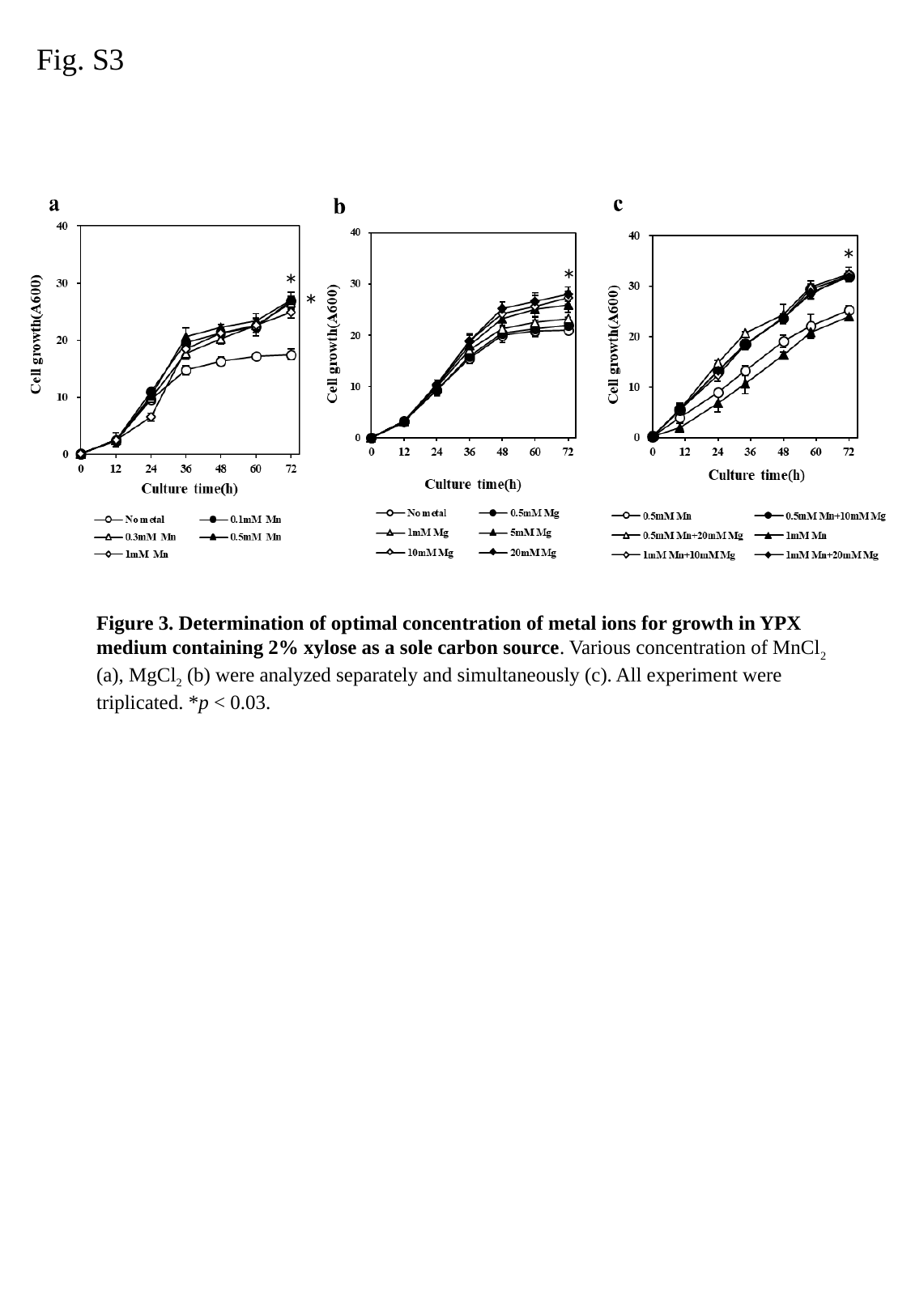

Fig. S3
*
*
*
*
Figure 3. Determination of optimal concentration of metal ions for growth in YPX medium containing 2% xylose as a sole carbon source. Various concentration of MnCl2 (a), MgCl2 (b) were analyzed separately and simultaneously (c). All experiment were triplicated. *p < 0.03.

## Slide 4
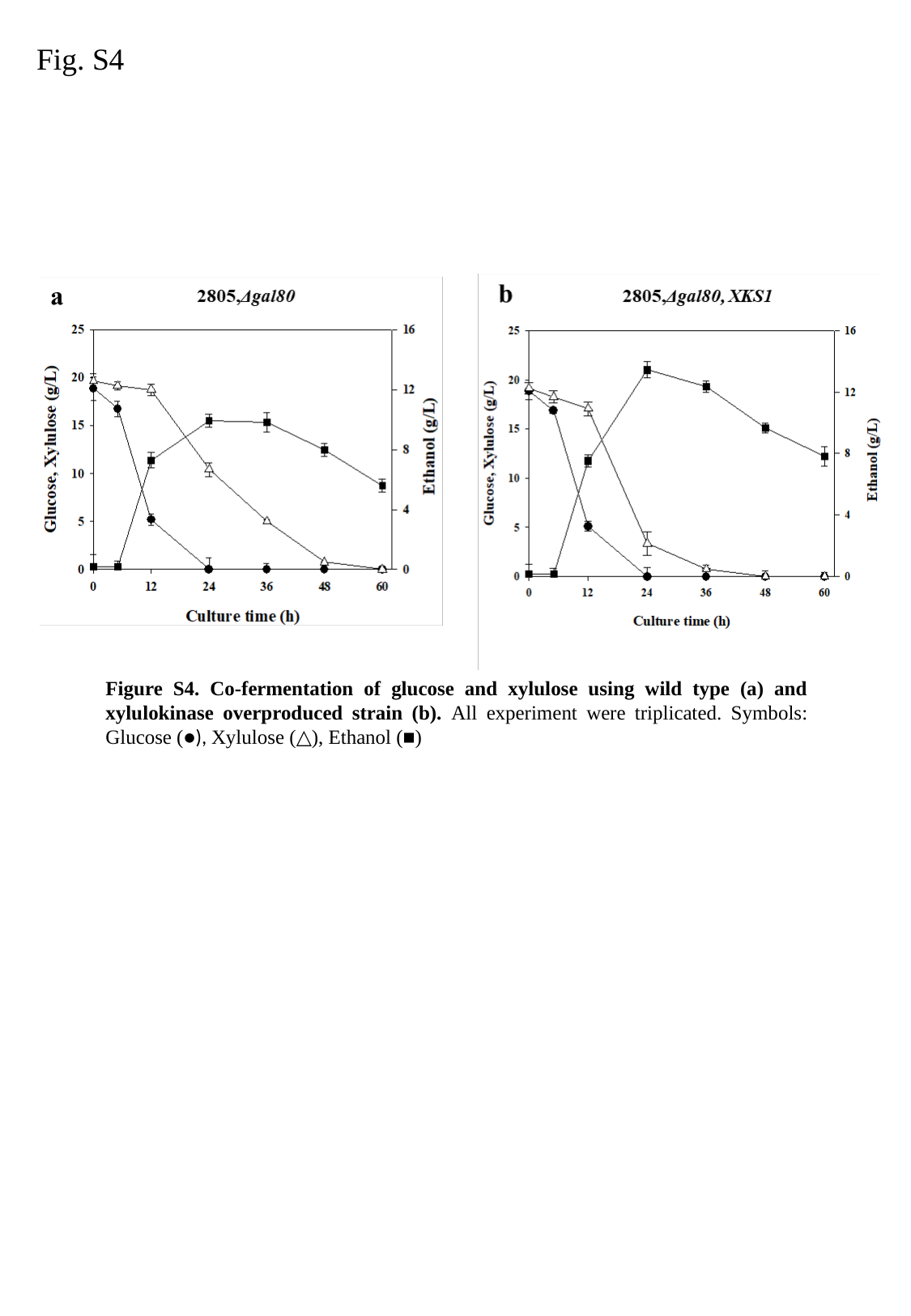

Fig. S4
Figure S4. Co-fermentation of glucose and xylulose using wild type (a) and xylulokinase overproduced strain (b). All experiment were triplicated. Symbols: Glucose (●), Xylulose (△), Ethanol (■)

## Slide 5
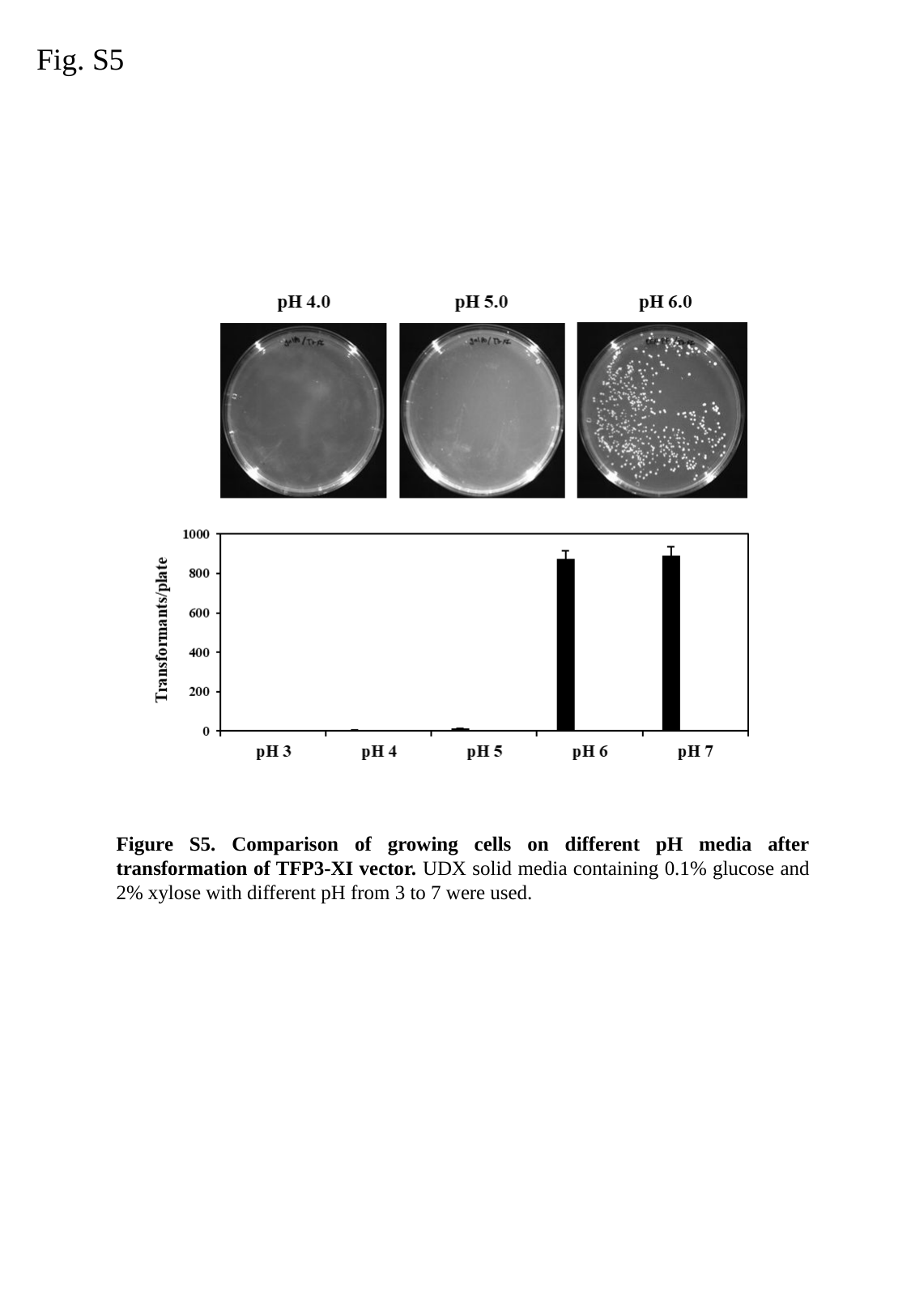

Fig. S5
Figure S5. Comparison of growing cells on different pH media after transformation of TFP3-XI vector. UDX solid media containing 0.1% glucose and 2% xylose with different pH from 3 to 7 were used.

## Slide 6
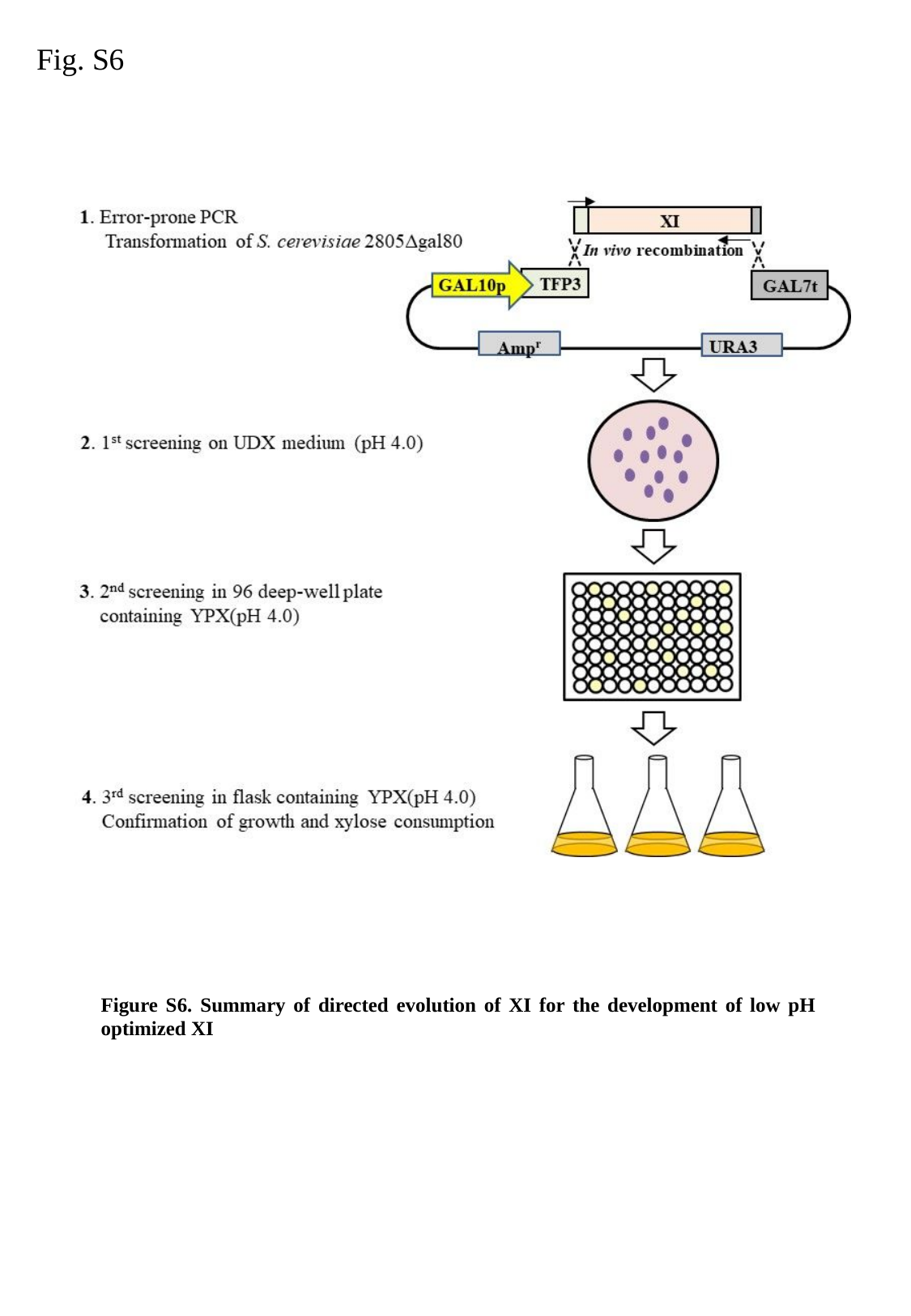

Fig. S6
Figure S6. Summary of directed evolution of XI for the development of low pH optimized XI
